# Disturbed Sleep is Not Good for the Heart: A Narrative Review

**DOI:** 10.2174/1573403X19666221130100141

**Published:** 2023-03-22

**Authors:** Meet Patel, Harshani Yarlagadda, Shubekshya Upadhyay, Ritesh Neupane, Umer Qureshi, Joseph D. Raco, Rahul Jain, Rohit Jain

**Affiliations:** 1 Department of Internal Medicine, Tianjin Medical University, Tianjin, P.R. China;; 2 Avalon University School of Medicine, Willemstad, Curaçao;; 3 Department of Public Health, University of California, Berkeley, CA, USA;; 4 Department of Internal Medicine, Penn State Milton S. Hershey Medical Center, Hershey, PA, USA;; 5 Penn State College of Medicine, Hershey, PA, USA

**Keywords:** Obstructive sleep apnea/pathophysiology, apnea-hypopnea index, congestive heart failure, continuous airway positive pressure, mandibular advancement device, hypoglossal nerve stimulation

## Abstract

Sleep-related breathing disorders, including obstructive sleep apnea (OSA) and central sleep apnea (CSA), have a major impact on cardiovascular function. It has shown an association with hypertension, coronary artery disease, cardiac arrhythmias, sudden cardiac death, and congestive heart failure (CHF). This review focuses on highlighting the relationship between sleep apnea and CHF. We discuss the underlying pathophysiology, which involves the mechanical, neurohormonal, and inflammatory mechanisms; in addition, the similarities and differentiating clinical features of OSA in patients with CHF and without CHF. We have also discussed several treatment strategies, including weight loss, continuous positive airway pressure (CPAP), supplemental oxygen therapy, theophylline, acetazolamide, mandibular advancement device, and hypoglossal nerve stimulation (HGNS). We conclude that since there are several overlapping clinical features in patients with OSA with Heart Failure (HF) and without HF, early detection and treatment are crucial to decrease the risk of HF, coronary artery disease, and stroke.

## 1 INTRODUCTION

Sleep apnea is a chronic medical condition characterized by repetitive arrests in breathing during sleep. There are two types of sleep apnea: obstructive sleep apnea (OSA) and central sleep apnea (CSA) [1]. Obstructive sleep apnea is caused by repeated upper airway muscle collapse during sleep, causing the chest muscles and diaphragm to work harder to open up the temporary blockage. Central sleep apnea is caused by altered respiratory drive from the CNS during sleep which causes insufficient ventilation [1]. The prevalence of OSA in the adult population is about 3%-7% in men and 2%-5% in women [2]. OSA is more common and more severe in males compared to females due to structural differences. Even though males tend to have a larger airway size compared to females, a male’s airway is more collapsible during breathing [3]. Risk factors associated with sleep apnea include obesity (BMI > 30), advanced age (peaks at approximately 55 years old), family history, craniofacial abnormalities, smoking, and alcohol consumption. An estimated 22 million Americans are currently affected by moderate to severe sleep apnea. OSA is believed to affect around 1 billion people around the world [4] (Table **1**).

Apnea-Hypopnea index (AHI) measures the number of apnea and hypopnea episodes per hour during sleep; a measure of severity of sleep apnea [4].

Congestive heart failure (CHF) is another chronic, progressive medical condition due to inefficient heart function; the abnormal heart muscle function causes insufficient blood flow throughout the heart and the body. Congestive heart failure affects about 23 million people around the globe, with about 5-6 million people in the United States alone [5]. There is a continued rise in the incidence and prevalence of CHF patients in the United States due to the elderly population and the available treatments to prolong patient’s lives [5]. The incidence is higher in men; however, the overall prevalence remains similar for both men and women [6]. It is the leading cause of hospitalization in individuals over the age of 65. This increasing number of individuals creates a significant strain on the healthcare system.

About 50% of all CHF patients have either central or obstructive sleep apnea disrupting the cardiovascular system and the sleep cycle [7]. The disordered breathing in OSA experienced by patients during sleep is the Cheyne-Stokes respiration [8]. The Cheyne-Stokes respiration is a specific form of breathing that consists of periods of apnea and hyperventilation in a cyclic pattern [9]. Multiple studies have shown this correlation. However, often sleep apnea goes undiagnosed causing the deterioration of cardiac function due to lack of management and treatment. The condition can increase the risk of heart failure by 140%, increase the risk of stroke by 60%, and increase the risk of coronary heart disease by 30% [10].

This paper focuses on highlighting the correlation between sleep apnea and congestive heart failure; in terms of pathophysiology and clinical outcome. In addition, the current treatment modalities available to improve the outcomes will also be discussed.

## 2 PATHOPHYSIOLOGY

Normal sleep cycle has various effects on the autonomic nervous system (ANA) and to understand the negative pathophysiological consequences of sleep apnea that may influence HF, the effects of the normal sleep cycle on ANA should be understood. Sleep cycle is divided into two distinct neurophysiological states - non-REM sleep (80%) and REM sleep (20%) [11].

Non-REM is divided into Stages 1, 2, 3, and 4. As the sleep deepens from stage 1 (Lightest) to stage 4 (Deepest), there is an increase in parasympathetic nervous activity and decrease in sympathetic nervous activity (SNA), resulting in decrease in blood pressure (BP) and heart rate (HR) [12]. During the period from wakefulness to stage 4 of non-REM sleep the SNA decline from 100 ± 9% to 41 ± 9%, BP from 90 ± 4 mm Hg to 80 ± 4 mm Hg and HR from 64 ± 2 beats per minute to 59 ± 2 beats per minute [13]. On the contrary, REM sleep is associated with loss of postural muscle tone which is an excitatory stimulus to SNA of muscles resulting in vasoconstriction of skeletal muscle blood vessels, inducing surges in BP and HR, and brief restoration of muscle tone during REM sleep (REM twitches) is associated with halting of SNA and surges in BP and HR. The mean level of SNA increased to 215 ± 11%. However, the average BP and HR remain similar to those during wakefulness levels [13]. A recent cross-sectional study found that REM- related OSA was prevalent in 56.3% of the study population and was more common in the mild to moderate severity subgroup of OSA [14].

OSA is characterized by atonia of genioglossus muscle during sleep, causing the tongue to fall backward and close the upper airway. In patients with OSA the pharynx is highly compliant and narrow that is prone to collapse upon normal withdrawal of pharyngeal dilator muscle tone at sleep onset, thus enabling airway narrowing (Hypopnea) or occlusion (apnea). Majority of the patients with OSA are obese and the deposition of fat adjacent to the pharynx is partly responsible for narrowing the pharyngeal lumen [7, 11, 15]. In addition to these mechanisms, evidence suggests that when lying down during nighttime, there is a redistribution of fluid accumulated during daytime in the legs to the upper parts of the body (Nocturnal Rostral fluid shift), which contributes to the distention of the neck veins and edema of parapharyngeal soft tissues, predisposing the individual to pharyngeal obstruction [16-20].

**Fig. (1):**
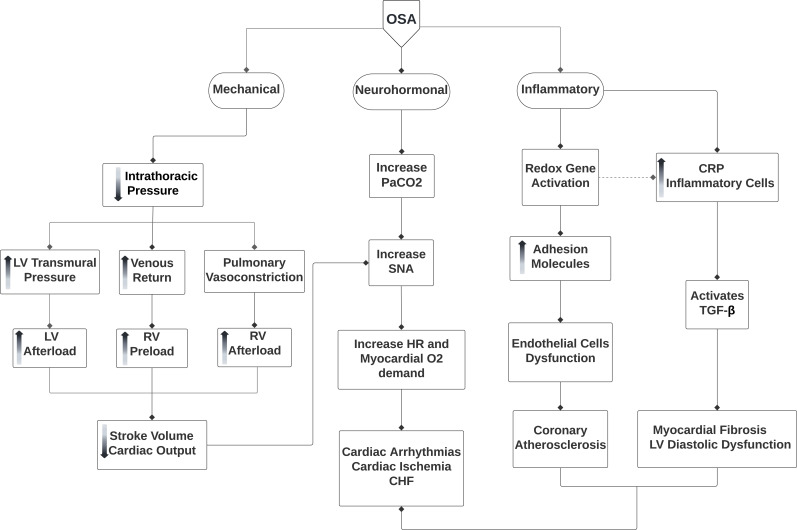
Pathophysiology of obstructive sleep apnea (OSA) leading to damage to the heart.

OSA affects the cardiovascular system through mechanical, neurohormonal and inflammatory mechanisms (Fig. **1**).

### 2.1 Mechinical

During the episode of OSA there is a generation of negative intrathoracic pressure (Intrathoracic pressure = visceral pleura pressure - thoracic pleura pressure) due to unavailing inspiratory efforts against obstructed pharynx. The drop in intrathoracic pressure results in an increase in venous return which increases right ventricle (RV) preload, resulting in RV distension and shifting of interventricular septum leftwards during diastole [21]. The latter disrupts left ventricular (LV) filling, and decreases LV preload, whereas drop in intrathoracic pressure increases LV transmural pressure (intracardiac pressure - intrathoracic pressure) and hence LV afterload [22, 23]. These factors act in conjunction to abate stroke volume and cardiac output (CO) [22].

### 2.2 Neurohormonal

OSA also amplifies SNA secondary to carbon dioxide retention [24], decreasing stroke volume and suppressing inhibitory effects on SNA by pulmonary stretch receptors. These effects of OSA on SNA contribute to increased LV afterload, heart rate and myocardial oxygen demand, which predispose the patients to a greater risk for cardiac arrhythmia, ischemia, LV hypertrophy and congestive heart failure [25]. Whereas the hypoxic and hypercarbia episodes induce pulmonary vasoconstriction, which increases RV afterload, making the patient vulnerable to right heart failure.

### 2.3 Inflammatory

Repeated cycles of apnea related hypoxia and post-apneic reoxygenation induce oxidative stress, generate reactive oxygen species and provokes inflammation. Patients with OSA have diminished Nitric oxide levels and hence impaired endothelial mediated vasodilation that contributes to the development of hypertension [26, 27]. Reactive oxygen species selectively activates inflammatory pathway by activating nuclear factor-kappa B (NF-κB), which leads to increased production of inflammatory mediators such as Interleukin (IL) - 6, IL-8, tumor necrosis factor-α and C-reactive protein [28] as well as adhesion molecules such as intracellular cell adhesion-1 (ICAM-1) and vascular cell adhesion molecules (VCAM-1), E selectin, and CD15, that facilitates endothelial damage and atherogenesis [29]. Infiltration of these inflammatory cells activates transforming growth factor-β, which induces differentiation of fibroblast to myofibroblast, causing myocardial fibrosis and LV diastolic dysfunction [30]. A randomized control trial demonstrated treatment of OSA with CPAP reduces carotid intima-media thickness, supporting the concept that OSA is an independent risk factor for atherosclerosis [31]. In addition, systemic review and meta-analysis conducted by Mi Lu and her colleagues provided imaging evidence supporting that patients with OSA are at higher risk of developing coronary atherosclerosis, cardiac remodeling and dysfunction and the severity of OSA is proportional to the grades of diastolic dysfunction and cardiac injury [32, 33]. Therefore, OSA is another mechanism that contributes to the development of HF.

## 3 DIAGNOSIS

A constellation of characteristic signs, symptoms, and radiographic findings often leads to the diagnosis of CHF. The signs and symptoms of CHF are related to the reduction of cardiac output or fluid retention. These include fatigue, dyspnea on exertion, orthopnea, cardiac wheezing, peripheral edema, crackles on auscultation, hepatic congestion and ascites, and laterally displaced apical pulsations. Elevated jugular venous pulsation is a sign of volume overload. Increased JVP correlates with increased pulmonary artery occlusion pressure. Chest X-ray (CXR) is used for differentiating cardiac causes from respiratory causes of dyspnea. CXR findings of cardiomegaly *i.e.* >50% cardiac to thoracic ratio, cephalization of blood vessels, increased interstitial markings and pleural effusions, are significant for heart failure. Similarly, brain natriuretic peptide (BNP) also helps to differentiate between cardiac and pulmonary causes of dyspnea. BNP is produced by ventricular muscle cells secondary to volume and pressure load. BNP > 100pg/ml is suggestive of heart failure with a sensitivity of over 90% and a predictive accuracy of 83% [34].

The clinical features of OSA in patients with HF are similar to those of OSA patients without HF, like difficulty maintaining sleep, nocturia, nocturnal dyspnea, lack of energy and fatigue [35]. Although they share various clinical features, they differ in some key aspects which include excessive daytime sleepiness (EDS) and obesity.

Most patients suffering from OSA with HF, when compared with general population, do not complain of EDS, despite significantly reduced sleep time and they have an Epworth Sleepiness Scale (ESS) score within normal limits (*i.e.* < 11), indicating that EDS is not a reliable indicator of the presence of OSA in the HF population [36]. The lack of EDS in these patients is associated with HF related increase in SNA [37].

Obesity has linear correlation with OSA [38], however, obesity is of less importance in the pathogenesis of OSA in patients with HF than in those without HF [36]. Therefore, in the HF population, the presence of obesity is insensitive to predicting the presence of OSA. Hence, factors other than obesity, like nocturnal rostral fluid displacement, as mentioned earlier must play an important role in the pathogenesis of OSA in the HF than in the general population [25]. The symptoms of sleep apnea experienced by a patient can vary; therefore, several therapies are available to improve the overall quality of their health.

## 4 TREATMENT

There are various treatment options available depending on the severity of sleep apnea. For milder cases, lifestyle changes such as a reduction in weight would be the primary recommendation. A decrease in weight by 7%-11% significantly improves OSA [39]. For moderate to severe cases, treatment can be divided as ventilation and medical.

### 4.1 Ventilation

The most common treatment method is a CPAP (Continuous Positive Airway Pressure) machine; this machine allows the upper airways to remain open to prevent apnea from occurring. CPAP therapy use has yielded a reduction in inflammatory biomarkers and an improvement in endothelial function [40]. A clinical trial of 20 patients who received nasal CPAP for 2 months showed improvement in their symptoms and a decrease in NF-kB and HIF-1a expression [41]. A retrospective cohort study found that patients with HF who received CPAP treatment for SA had a 90% two-year probability of survival compared to the 70% in undiagnosed and untreated individuals [42]. CPAP therapy showed a significant improvement in left ventricular ejection fraction (LVEF) in a three-month randomized controlled trial; this improvement was more notably evident in CHF patients with an LVEF < 30% [43]. In patients with OSA and HF, one month of CPAP therapy has been shown to increase the LVEF from 37% to 49% and reduce the daytime systolic blood pressure levels from 126 ± 6 to 116 ± 5 mmHg [44]. The nightly use of CPAP can reduce the daytime systolic BP by inhibiting the muscle sympathetic nerve activity, preventing vasoconstriction [45]. CPAP also reduced the occurrence of premature nocturnal ventricular beats [44].

The therapeutic effects of CPAP therapy are comparable to chronic usage of B-blockers in HF [7]. Beta-blockers work to inhibit the excess sympathetic activity of the heart and help to overall improve survival in patients suffering from HF [44]. The addition of carvedilol for about 10.4 months showed a 35% decrease in the rate of death and a 24% decrease in hospitalization in patients with severe chronic heart failure [46].

Supplemental oxygen given at night has shown a reduction in AHI by 50% in individuals with CSA and HF [44]. Oxygen administration has not shown any improvement in LVEF or cardiac function. Consequently, oxygen can increase the generation of free radicals, which can lead to oxidative stress. This type of stress can increase blood pressure and LV filling pressure, leading to lower cardiac output [44].

A mandibular advancement device (MAD) is a device that temporarily moves the tongue and jaw forward to prevent the collapse of the pharyngeal airway. The device is either custom-made or semi-custom to correctly fit in a patient’s mouth. MAD is contraindicated for patients with dentures or dental issues. It is also known as a mandibular advancement splint (MAS). At times, the devices are preferred by patients since they are easily portable and user-friendly compared to traditional CPAP machines. A randomized, crossover trial showed that 4 weeks of MAS therapy improved daytime sleepiness [47]. The same trial showed that 23% of the 85-person sample population reported no snoring symptoms with the use of MAS [47]. The patient compliance to use these devices was higher at 99%; the patients were more willing to continue with this treatment method [47].

**Fig. (2):**
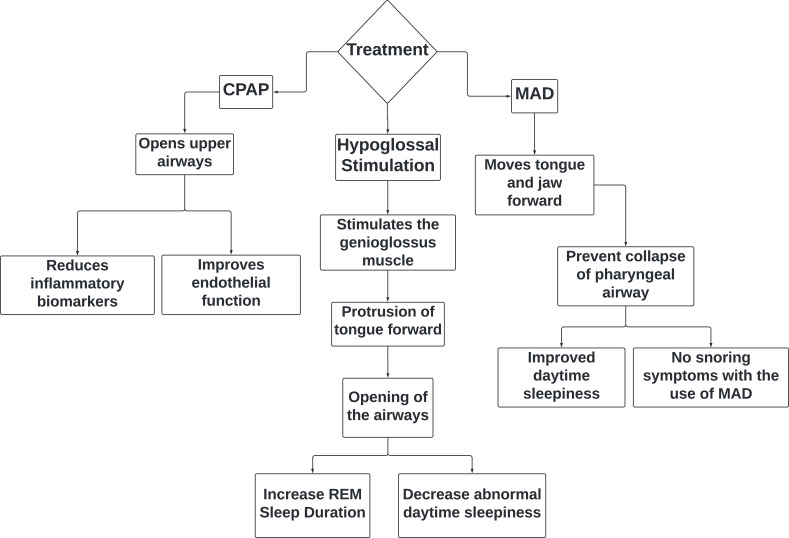
Treatment options for obstructive sleep apnea and their effect.

Hypoglossal nerve stimulation (HGNS) is a newer treatment modality that has exhibited a reduction in the apnea-hypopnea index scores by 68% within 12 months of stimulator implantation [48]. This procedure is most effective in patients with a BMI less than 32 and who have been unresponsive to CPAP therapy. The electrical stimulation of an airway dilator muscle known as the genioglossus muscle allows for the protrusion of the tongue [49]. This action has caused a therapeutic effect in patients with OSA by opening up their airways. HGNS has shown improvement in the OSA-associated symptoms and severity of OSA [49]. This improvement included a longer duration of REM sleep and decreased abnormal daytime sleepiness (Fig. **2**).

### 4.2 Medical

Theophylline is a medication that inhibits type III and type IV phosphodiesterase (PDE), which allows for smooth muscle relaxation, resulting in bronchodilation. It also works to block adenosine to increase cardiac contractility. A randomized trial consisting of 15 patients who suffer from both CSA and HF had a reduction in AHI but no improvement in LVEF when given theophylline for 5 days [44]. This therapy showed a decrease in the number of episodes of apnea and hypopnea in a span of an hour of sleep [50]. However, theophylline is no longer recommended due to its adverse effects, including cardiac arrhythmias and sudden cardiac death. Acetazolamide is a carbonic anhydrase inhibitor that stimulates breathing by inducing metabolic acidosis. A randomized trial of 12 patients who had both CSA and HF, the administration of acetazolamide 1 hour before bed for 6 nights, showed a reduction in AHI by 38% [51]. The short-term single dose of acetazolamide showed an improvement in daytime sleepiness and fatigue in both OSA and CSA [52]. Presently, it is not recommended as a treatment option; more studies need to be conducted to determine what long-term effect of the medication has on safety and efficacy [44].

## CONCLUSION

The sleep-related breathing condition known as sleep apnea significantly affects the sleep cycle and the cardiovascular system of those who suffer from it. The incidence of patients with OSA and CHF is expected to increase as the population ages. To ensure that patients OSA and CHF receive adequate medical care, it is crucial to understand the importance of screening and diagnosing patients with these conditions. Currently, there are several treatment options, with CPAP therapy being the first line for a large number of patients. Researchers are also exploring new treatment methods to increase the clinical outcome and quality of life in all OSA patients. OSA patients with HF share many of the same clinical characteristics as patients without HF, therefore accurate diagnosis and early detection are important to prevent damage to the cardiovascular system and decrease the risk of heart failure, stroke, and coronary artery disease. Consequently, the screening assessment of sleep apnea is highly recommended in patients with conditions like hypertension, diabetes, obesity, and dyslipidemia. The additional referrals to sleep specialists and cardiologists should be considered accordingly depending on each patient’s clinical severity to help contribute to their long-term prognosis and treatment plan.

## AUTHOR’S CONTRIBUTIONS

Meet Patel, Harshani Yarlagadda, Umer Qureshi and Joseph D. Raco assisted in article concept and design, acquisition of data, drafting of the manuscript, and final approval. Shubekshya Upadhyay, Ritesh Neupane, Rahul Jain and Rohit Jain assisted in the article concept and design, analysis and interpretation of the data, and revision of the manuscript for important intellectual content and final approval. Meet Patel, Harshani Yarlagadda, Shubekshya Upadhyay, Ritesh Neupane and Rohit Jain further assisted in revisions of the final manuscript.

## Figures and Tables

**Table 1:** Differentiating criteria for the severity of sleep apnea based on apnea-hypopnea index.

**Severity of Sleep Apnea**	**Apnea-Hypopnea Index (AHI)**
**Mild**	5-15 per hour
**Moderate**	> 15 - 30 per hour
**Severe**	> 30 per hour

## LIST OF ABBREVIATIONS

CHF: Congestive Heart Failure
CPAP: Continuous Positive Airway Pressure
CSA: Central Sleep Apnea
HGNS: Hypoglossal Nerve Stimulation
OSA: Obstructive Sleep Apnea

## References

[1] Jun J.C., Chopra S., Schwartz A.R. (2016). Sleep apnoea.. Eur. Respir. Rev..

[2] Lurie A. (2011). Obstructive sleep apnea in adults: epidemiology, clinical presentation, and treatment options.. Adv. Cardiol..

[3] Mohsenin V. (2003). Effects of gender on upper airway collapsibility and severity of obstructive sleep apnea.. Sleep Med..

[4] Malhotra A., Ayappa I., Ayas N. (2021). Metrics of sleep apnea severity: beyond the apnea-hypopnea index.. Sleep.

[5] Khattak H.K., Hayat F., Pamboukian S.V., Hahn H.S., Schwartz B.P., Stein P.K. (2018). Obstructive sleep apnea in heart failure: Review of prevalence, treatment with continuous positive airway pressure, and prognosis.. Tex. Heart Inst. J..

[6] Strömberg A., Mårtensson J. (2003). Gender differences in patients with heart failure.. Eur. J. Cardiovasc. Nurs..

[7] Bradley T.D., Floras J.S. (2003). Sleep apnea and heart failure: Part I: obstructive sleep apnea.. Circulation.

[8] Schulz R., Blau A., Börgel J. (2007). Sleep apnoea in heart failure.. Eur. Respir. J..

[9] Rudrappa M., Modi P., Bollu P.C. (2022). Cheyne Stokes Respirations.StatPearls, StatPearls Publishing Copyright © 2022..

[10] Jean-Louis G., Zizi F., Clark L.T., Brown C.D., McFarlane S.I. (2008). Obstructive sleep apnea and cardiovascular disease: role of the metabolic syndrome and its components.. J. Clin. Sleep Med..

[11] Javaheri S., Javaheri S., Javaheri A. (2013). Sleep apnea, heart failure, and pulmonary hypertension.. Curr. Heart Fail. Rep..

[12] Trinder J., Kleiman J., Carrington M. (2001). Autonomic activity during human sleep as a function of time and sleep stage.. J. Sleep Res..

[13] Somers V.K., Dyken M.E., Mark A.L., Abboud F.M. (1993). Sympathetic-nerve activity during sleep in normal subjects.. N. Engl. J. Med..

[14] Arjun P., Nair S.C., Azeez A., Nair S. (2022). Proportion of rapid eye movement sleep related obstructive sleep apnea (REM related OSA) in patients with sleep disordered breathing: A cross sectional study.. Lung India.

[15] Ryan C.M., Bradley T.D. (2005). Pathogenesis of obstructive sleep apnea.. J. Appl. Physiol..

[16] Yumino D., Redolfi S., Ruttanaumpawan P. (2010). Nocturnal rostral fluid shift: a unifying concept for the pathogenesis of obstructive and central sleep apnea in men with heart failure.. Circulation.

[17] Chiu K.L., Ryan C.M., Shiota S. (2006). Fluid shift by lower body positive pressure increases pharyngeal resistance in healthy subjects.. Am. J. Respir. Crit. Care Med..

[18] Redolfi S., Yumino D., Ruttanaumpawan P. (2009). Relationship between overnight rostral fluid shift and obstructive sleep apnea in nonobese men.. Am. J. Respir. Crit. Care Med..

[19] Shiota S., Ryan C.M., Chiu K.L. (2007). Alterations in upper airway cross-sectional area in response to lower body positive pressure in healthy subjects.. Thorax.

[20] Su M.C., Chiu K.L., Ruttanaumpawan P. (2008). Lower body positive pressure increases upper airway collapsibility in healthy subjects.. Respir. Physiol. Neurobiol..

[21] Brinker J.A., Weiss J.L., Lappé D.L. (1980). Leftward septal displacement during right ventricular loading in man.. Circulation.

[22] Orban M., Bruce C.J., Pressman G.S. (2008). Dynamic changes of left ventricular performance and left atrial volume induced by the mueller maneuver in healthy young adults and implications for obstructive sleep apnea, atrial fibrillation, and heart failure.. Am. J. Cardiol..

[23] Bradley T.D., Hall M.J., Ando S., Floras J.S. (2001). Hemodynamic effects of simulated obstructive apneas in humans with and without heart failure.. Chest.

[24] Somers V.K., Mark A.L., Zavala D.C., Abboud F.M. (1989). Influence of ventilation and hypocapnia on sympathetic nerve responses to hypoxia in normal humans.. J. Appl. Physiol..

[25] Kasai T., Bradley T.D. (2011). Obstructive sleep apnea and heart failure: pathophysiologic and therapeutic implications.. J. Am. Coll. Cardiol..

[26] Ip M.S.M., Lam B., Chan L.Y. (2000). Circulating nitric oxide is suppressed in obstructive sleep apnea and is reversed by nasal continuous positive airway pressure.. Am. J. Respir. Crit. Care Med..

[27] Carlson J.T., Rångemark C., Hedner J.A. (1996). Attenuated endothelium-dependent vascular relaxation in patients with sleep apnoea.. J. Hypertens..

[28] Shamsuzzaman A.S.M., Winnicki M., Lanfranchi P. (2002). Elevated C-reactive protein in patients with obstructive sleep apnea.. Circulation.

[29] Garvey J.F., Taylor C.T., McNicholas W.T. (2009). Cardiovascular disease in obstructive sleep apnoea syndrome: The role of intermittent hypoxia and inflammation.. Eur. Respir. J..

[30] Westermann D., Lindner D., Kasner M. (2011). Cardiac inflammation contributes to changes in the extracellular matrix in patients with heart failure and normal ejection fraction.. Circ. Heart Fail..

[31] Drager L.F., Bortolotto L.A., Figueiredo A.C., Krieger E.M., Lorenzi-Filho G. (2007). Effects of continuous positive airway pressure on early signs of atherosclerosis in obstructive sleep apnea.. Am. J. Respir. Crit. Care Med..

[32] Lu M., Wang Z., Zhan X., Wei Y. (2021). Obstructive sleep apnea increases the risk of cardiovascular damage: A systematic review and meta-analysis of imaging studies.. Syst. Rev..

[33] Raut S., Gupta G., Narang R. (2021). The impact of obstructive sleep apnoea severity on cardiac structure and injury.. Sleep Med..

[34] Figueroa M.S., Peters J.I. (2006). Congestive heart failure: Diagnosis, pathophysiology, therapy, and implications for respiratory care.. Respir. Care.

[35] Javaheri S. Basics of Sleep Apnea and Heart Failure 2013.. https://www.acc.org/latest-incardiology/articles/2014/07/22/08/25/basics-of-sleep-apnea-andheart-failure.

[36] Arzt M., Young T., Finn L. (2006). Sleepiness and sleep in patients with both systolic heart failure and obstructive sleep apnea.. Arch. Intern. Med..

[37] Taranto Montemurro L., Floras J.S., Millar P.J. (2012). Inverse relationship of subjective daytime sleepiness to sympathetic activity in patients with heart failure and obstructive sleep apnea.. Chest.

[38] Jehan S., Zizi F., Pandi-Perumal S.R. (2017). Obstructive sleep apnea and obesity: Implications for public health.. Sleep Med. Disord..

[39] Tham K.W., Lee P.C., Lim C.H. (2019). Weight management in obstructive sleep apnea.. Sleep Med. Clin..

[40] Romero-Corral A., Caples S.M., Lopez-Jimenez F., Somers V.K. (2010). Interactions between obesity and obstructive sleep apnea: Implications for treatment.. Chest.

[41] Lu D., Li N., Yao X., Zhou L. (2016). Potential inflammatory markers in obstructive sleep apnea-hypopnea syndrome.. Bosn. J. Basic Med. Sci..

[42] Javaheri S., Caref E.B., Chen E., Tong K.B., Abraham W.T. (2011). Sleep apnea testing and outcomes in a large cohort of Medicare beneficiaries with newly diagnosed heart failure.. Am. J. Respir. Crit. Care Med..

[43] Egea C.J., Aizpuru F., Pinto J.A. (2008). Cardiac function after CPAP therapy in patients with chronic heart failure and sleep apnea: A multicenter study.. Sleep Med..

[44] Arzt M., Bradley T.D. (2006). Treatment of sleep apnea in heart failure.. Am. J. Respir. Crit. Care Med..

[45] Usui K., Bradley T.D., Spaak J. (2005). Inhibition of awake sympathetic nerve activity of heart failure patients with obstructive sleep apnea by nocturnal continuous positive airway pressure.. J. Am. Coll. Cardiol..

[46] Packer M., Coats A.J.S., Fowler M.B. (2001). Effect of carvedilol on survival in severe chronic heart failure.. N. Engl. J. Med..

[47] Gotsopoulos H., Chen C., Qian J., Cistulli P.A. (2002). Oral appliance therapy improves symptoms in obstructive sleep apnea: A randomized, controlled trial.. Am. J. Respir. Crit. Care Med..

[48] Maresch K.J. (2018). Hypoglossal nerve stimulation: effective longterm therapy for obstructive sleep apnea.. AANA J..

[49] Eastwood P.R., Barnes M., Walsh J.H. (2011). Treating obstructive sleep apnea with hypoglossal nerve stimulation.. Sleep.

[50] Javaheri S., Parker T.J., Wexler L., Liming J.D., Lindower P., Roselle G.A. (1996). Effect of theophylline on sleep-disordered breathing in heart failure.. N. Engl. J. Med..

[51] Javaheri S. (2006). Acetazolamide improves central sleep apnea in heart failure: a double-blind, prospective study.. Am. J. Respir. Crit. Care Med..

[52] Schmickl C.N., Landry S.A., Orr J.E. (2020). Acetazolamide for OSA and central sleep apnea.. Chest.

